# Decreased serum level of soluble-leptin-receptor in patients with systemic lupus erythematosus

**Published:** 2012-09-30

**Authors:** K Bagheri, P Ebadi, S Naeimi

**Affiliations:** 1 Department of Immunology, Faculty of medicine, Kazerun Branch, Islamic Azad University, Kazerun, Iran; 2 Department of Biochemistry, Faculty of medicine, Kazerun Branch, Islamic Azad University, Kazerun, Iran

**Keywords:** Soluble-leptin-receptor, Autoantibody, Systemic lupus erythematosus, Depression

## Abstract

**Background:**

There is some evidence suggesting that leptin and its negative regulator, soluble-leptinreceptor (SLR) may be able to influence inflammatory and autoimmune processes.

**Methods:**

In this study, several variables including socio-demographics, health-related habits, depression score, serum molecules and blood parameters besides the SLR level were evaluated in patients with SLE (SLE-patients) and healthy controls.

**Results:**

The patients had significantly lower SLR level and higher depression score than the controls and both of these variables have a significant association with the occurrence of disease in logistic regression model. Moreover, the results of Pearson correlation analysis showed that patients’ SLR level was negatively correlated with their weights and BDI scores.

**Conclusion:**

For the first time, this study indicated a lower level of SLR in SLE-patients and suggested that lower concentrations of SLR in these patients may be implicated in the pathogenesis of SLE.

## Introduction

It is revealed that leptin could promote inflammatory and autoimmune processes (1). SLR is the extracellular part of the leptin receptor which may act as a negative regulator of leptin activity (2) and may influence pro-inflammatory responses in some autoimmune disorders like SLE. SLE as a prototype of multisystemic autoimmune diseases has been recognized for decades. However, the exact etiopathogenesis of SLE is still speculative and controversial, it has been demonstrated that many variables may be involved in the etiopathology of SLE. An abnormal SLR level may also have an effect on the pathogenesis of SLE.

There are several contradictory reports on leptin in rheumatic diseases. For example, although some authors have reported that leptin levels of SLE-patients (3) and rheumatoid arthritis (RA)-patients (4) are higher than those of normal controls, some others have found similar leptin plasma levels between SLE-patients (5) and RA-patients (6) with their respective controls. The pattern of change in leptin and SLR concentrations varies in different conditions, is often reverse and sometimes it is independent and unbiased by each other. Therefore, considering the role of SLR in leptin regulation, it is interesting to know whether or not SLR level of SLE-patients is statistically different from normal. This study also assessed whether this SLR level is correlated with other variables such as smoking habits, socio-demographic, psychological, environmental, dietary, laboratory, and lifestyle parameters which may be involved in the etiopathology of SLE.

## Materials and Methods 

### SLE-patients and control group

We studied 34 unrelated SLE-patients which were not under therapy. Healthy sex and age matched controls were also randomly selected. Informed consent was obtained from all the participants.

### Detection of ANA, anti-dsDNA antibody and complement levels

The presence of ANA and anti-dsDNA antibodies was determined by ELISA (Aesku.lab, Aesku Diagnostics, Wendelsheim,Germany). Plasma concentrations of the Complement C3 and C4 component were quantified in nephelometry.

### Detection of serum C-reactive-protein (CRP) and Rheumatoid factor (RF)

The acute-phase inflammatory response was assessed by determining the serum level of CRP in a high-sensitive approach (HS-CRP). The serological status for RF was performed using a semi-quantitative kit.

### Hematological studies

A complete blood count (CBC) with differential was done on the hematology counter (Coulter Sysmex K1000 machine).

### Assessment of depression status

Depression score was measured using the Persian translated and validated version of the Beck's depression Inventory (BDI).7 The obtained BDI-scores were used for all statistical analysis.

### Questionnaires

A team of interviewers, who received training, completed questionnaires consisting of variables, all of which were defined as quantitative values. These variables were contain socio-demographic and lifestyle parameters [including age, weight, educational level, tobacco smoking rate and duration, physical activity, consumption of contraceptive pills, hair products, alcohol, deodorant or air-freshener, sunscreen creams and lotions dietary habits, sunlight exposure, contact with pesticide or insecticide poisons, detergent or chemical solvents]. All the discussed variables in the questionnaires along with all the measured factors and laboratory parameters were 50 variables which were classified into 2 set of main variables (blood factors and non-blood factors).

### Measurement of SLR level

The serum SLR levels were assessed in triplicate by a commercial human specific sandwich ELISA kit (Quantikine, R&D Systems Inc, Minneapolis, MN, USA), according to the manufacturer’s guidelines. The minimum detectable dose (MDD) for this kit ranged from 0.020-0.128 ng/ml.

### Statistical analysis

The statistical significance of the differences between cases and controls was calculated by GLM multivariate analysis. Moreover, the impact of all main variables on the occurrence of disease was analyzed using binary logistic regression analysis, in order to assess odds ratio (OR) for the risk of systemic lupus erythematosus. Furthermore, the statistical correlation between variables was shown by Pearson correlation test. Data analysis was carried out using spss version 17 software and significant levels were considered 0.05. To illustrate differences between patients and controls and how the covariates were positively or negatively correlated we used a box plot and scotter plot with fitted regression lines respectively.

## Results

**Fig. 1:**
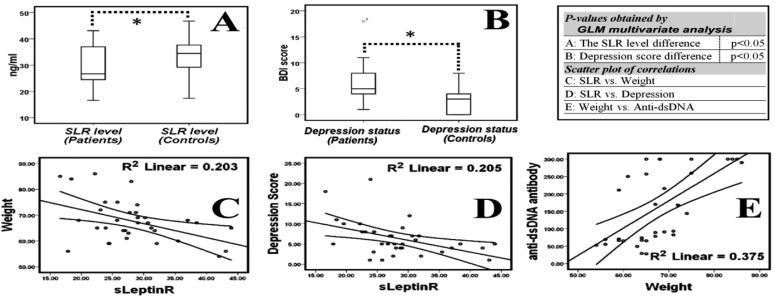
Box plot comparison and scatter plot relationships of some important variables.

However, by using a GLM multivariate analysis, there were no significant (P>0.05) differences for socio-demographic or lifestyle parameters [including tobacco smoking rate and duration, physical activity, alcohol consumption, dietary habits and etc.] between groups, but patients had a higher depression score (P=0.009) than controls (Table 1, Figure 1B). We also found that although there was no significant difference between their weights, the patients had significantly lower (P=0.026) SLR level than controls (Table 1, Figure 1A). GLM multivariate analysis demonstrated that patients have a significantly lower level of complement component (C3 and C4) and Hb-Hct but higher level of ANA, anti-dsDNA, CRP, RF, ESR1h and ESR2h than the controls (Table 1).

**Table 1:** Comparison of all blood markers and some non-blood variables in SLE-patients and healthy control group

Variables		Lupus Patients		Healthy Controls		P-value
		Mean ±SE**	(Range)	Mean ±SE	(Range)	
Auto-antibodies	ANA (index values)	4.89±0.28	(1.24-7.45)	0.5±0.08	(0.2-1.18)	0.000*
	Anti-dsDNA (U/ml)	161.5±17.97	(28-300)	7.46±1.4	(3.1-18.5)	0.000*
Complement system	C3 (g/L)	1.08±0.05	(0.34-1.58)	1.49±0.08	(0.98-1.86)	0.000*
	C4 (g/L)	0.2±0.01	(0.06-0.36)	0.3±0.1	(0.21-0.35)	0.001*
CRP (mg/L)		8.37±1.25	(2.3-37.84)	1.86±0.15	(1.14-3.04)	0.003*
RF		0.32±0.1	(0-2)	0	(0)	0.055
Erythrocyte-sedimentation-rate (ESR)-1hrs (mm/hr)		18.58±2.05	(5-61)	6.92±0.9	(3-14)	0.001*
ESR-2hrs (mm/hr)		36.08±3.01	(8-86)	13.92±1.55	(7-25)	0.000*
Hematological studies	Leukocyte (counts×1000/µL)	6.79±0.33	(3.15-10.5)	6.58±0.3	(5.1-9.4)	0.716
	Erythrocyte (counts×1000000/µL)	4.63±0.11	(3.12-5.58)	4.84±0.12	(4.08-5.45)	0.278
	Platelet (counts×1000/µL)	253.15±9.73	(160-398)	289.46±15.19	(202-383)	0.054
	Hemoglobin (Hb) (g/dL)	12.31±0.23	(9.3-14.9)	14.53±0.5	(12.5-17.8)	0.000*
	Hematocrit (Hct) (%)	37.9±0.66	(28.8±43.6)	43.83±1.12	(39.2-51.7)	0.000*
	Neutrophil (%)	53.88±1.63	(38-74)	50.53±1.21	(41-55)	0.232
	Lymphocyte (%)	42.14±1.69	(23-62)	44.38±1.37	(37-54)	0.441
	Monocyte (%)	2.29±0.26	(1-6)	3.23±0.41	(1-6)	0.064
	Eosinophil (%)	1.35±0.08	(1-2)	1.53±0.24	(1-4)	0.360
SLR level (ng/ml)		28.35±1.16	(16.59-43.98)	33.69±2.16	(17.37-46.74)	0.026*
Depression score (BDI score)		6.38±0.75	(1-21)	2.84±0.74	(0-8)	0.009*
Weight (Kg)		67.58±1.40	(54-86)	65.53±1.28	(56-72)	0.401

^*^The P values <0.05 were considered as statistically significant

^**^The results were expressed as mean ± standard error of the mean (Mean ±SE) with the range values (minimum and maximum) of the obtained data from separate groups.

Odds ratios and 95% confidence intervals were also calculated for 2 set of main variables (blood factors and non-blood factors) (Table 2). Decreased serum level of SLR showed a significant association with the occurrence of disease in logistic regression model. Between all the above blood factors, the level of C3, C4, CRP, ESR1h, ESR2h and hemoglobin were significantly correlated to the occurrence of disease which only the level of complement component (C3 and C4) and hemoglobin, like our new proposed marker (SLR) showed a significant negative correlation with the occurrence of systemic lupus erythematosus. The logistic regression analysis for all non blood factors showed that only the BDI score of depression was significantly related to the occurrence of disease and may be a risk factor for development of lupus. Moreover, the results of Pearson correlation analysis between all variables showed some interesting results which could be confirm by previous studies. For example the patients’ BDI score was correlated positively with tobacco consumption rate (r=+0.551, P=0.001) or duration (r=+0.506, P=0.002) and ANA titer (r=+0.518, P=0.002) and negatively with SLR level (r= -0.453, P=0.007). On the other hands, patients’ weight was correlated negatively with their SLR level (r= -0.451, P=0.007) and positively with their anti-dsDNA titer (r=+0.612, P=0.000). We used box plot to show differences between patients and controls which 2 important variables shown in figure 1 (A and B), were significantly different by positively with their anti-dsDNA titer (r=+0.612, P=0.000). We used box plot to show differences between patients and controls which 2 important variables shown in figure 1 (A and B), were significantly different by GLM multivariate analysis. Scatter plots shown in figure were also drawn and overlaid with fitted regression lines (with 95% confidence intervals) to illustrate how the plotted parameters (C, D and E) were positively or negatively correlated.

**Table 2:** The binary logistic regression analysis for all main non-blood and blood variables in order to assess the impact of them on the occurrence of disease.

Non-blood factors	Odds ratio	95% CI		P-value
		Lower	Upper	
Age (years)	1.054	0.955	1.164	0.292
Weight (Kg)	1.042	0.948	1.146	0.394
Smoking rate (the average number)	1.013	0.342	3.000	0.981
Duration of smoking (months)	0.995	0.956	1.036	0.802
Sunlight exposure (minutes of direct contact per day)	1.018	0.911	1.137	0.756
Depression score (BDI scores)	1.453	1.087	1.941	0.012*
Physical activity (minutes per week)	1.000	0.981	1.020	0.967
High fat food (servings per week)	0.982	0.797	1.209	0.864
**Blood markers**	**Odds ratio**	**95% CI**		**P-value**
		**Lower**	**Upper**	
C3 (g/L)	0.010	0.000	0.213	0.003*
C4 (g/L)	0.000	0.000	0.003	0.005*
CRP (mg/L)	30.841	2.239	424.816	0.010*
SLR level (ng/ml)	0.901	0.819	0.992	0.034*
ESR1h (mm/hr)	1.489	1.142	1.940	0.003*
ESR2h (mm/hr)	1.241	1.083	1.422	0.002*
Leukocyte (counts×1000/µL)	1.074	0.737	1.565	0.709
Hemoglobin (g/dL)	0.317	0.144	0.701	0.005*
Neutrophil (%)	1.052	0.968	1.142	0.230
Lymphocyte (%)	0.970	0.899	1.047	0.433
Monocyte (%)	0.678	0.445	1.035	0.072

^*^The P values <0.05 were considered as statistically significant

## Discussion

Patients’ lower level of C3 and C4 complement component and their negative significant association (Odds Ratios) with the occurrence of disease which respectively were shown by GLM multivariate and logistic regression analysis could be as a result of more activation of complement in these patients. On the other hands higher level of other blood factors such as CRP, ESR1h, ESR2h and etc in patient group and their relative risk (Odds Ratios) for SLE disease can often be as a result of some inflammatory and autoimmune events during the diseases' process. For example in the presence of inflammation, the liver rapidly increases its synthesis of fibrinogen resulting in an elevated ESR in these patients. The significant lower of Hb or Hct level in our patient group in GLM multivariate analysis and their respective odds Ratios for disease is believed to be due to bone marrow depression resulting from kidney involvement. This is in harmony with the view of Bauer who reported that a mild to moderate normochromic microcytic anemia is seen in almost every patient with SLE (8-10).

Laboni et al. reported a mild to moderate depression (mean BDI score=15.8+/-9.9) in lupus patients as their study group with disabling tiredness (11). It is also reported that several mood and anxiety disorders are more common in women with SLE compared with the general population (12). In this study, we demonstrated that depression BDI score in the patients were higher than those of the controls. We also showed that higher BDI score, as a probable risk factor, is correlated with the occurrence of lupus disease in logistic regression analysis. Our results are consistent with some of the reports such as the above-referenced study despite the lowering of our average depression score compared with some reports. This lowering could be related to many other variables, including cultural contexts, personality and habit differences. Incidentally, in this study we think that some habits like, alcoholism, cigarette smoking and hookah consumption may be a cause of higher depression score in lupus patients. In this regard, new reported evidence is consistent with the conclusion that there is a cause and effect relationship between smoking and depression in which cigarette smoking increases the risk of symptoms of depression (13). We also showed that more depressed SLE-patients had higher levels of ANA autoantibodies. This finding could be explained due to the observation that autoantibodies play an important role in the pathogenesis of depression. It is suggested that depression can be regarded as autoimmune disease caused by various autoantibodies. For example, it had been shown that major depression may be accompanied by systemic immune activation or an inflammatory response with higher autoantibody titers (14).

We also found that patients had significantly lower SLR level than controls. Moreover, lower level of this blood marker was negatively associated with the occurrence of disease in logistic regression model. It means that SLR level may be a diagnostic or predictive factor in lupus patients. There are several contradictory reports on leptin in rheumatic diseases (3-6). Leptin has also proven to be a factor that contributes to renal diseases (15,16). Furthermore, renal involvement remains a major determinant of the outcome in SLE. Recently, it has been clear that the SLR also neutralizes leptin. It also serves as antagonists of the transport of leptin (17). Therefore, considering the role of SLR in leptin regulation, it seems likely that lower concentrations of SLR in these patients may be implicated in the pathogenesis of lupus-related renal disease. Further investigations are required to explain the potential influence of SLR on the disease outcome in SLE-patients.

We also showed a negative correlation between weight and SLR level which was in line with most of the recent findings. For example, it is reported that the reduction of SLR concentrations in overweight and obese individuals may be the result of an increase in circulating leptin (18). However, Ogawa T et al. showed that the SLR level is negatively correlated with serum leptin level (19). In our study, the negative correlation of serum SLR and patient’s weight or depression score, together with its lower level in SLE-patients with a positive association with disease occurrence indicate that SLR level may have an effect on the pathogenesis of this autoimmune disease.

We also showed that higher weight is associated with higher anti-dsDNA titer in SLE-patients. There are some consistent results in this regard. For example, it was revealed that obese SLE-patients had worse functional capacity, more fatigue, and higher concentrations of inflammation markers (20). Finally, we determined several risk factors for SLE including overweight, depression and smoking.

Indeed, as a new result, we found that serum level of SLR was significantly lower in SLE-patients than controls and may be a diagnostic or predictive risk factor in lupus patients. To our knowledge, this study is also relatively novel in that it simultaneously examines the relationship between serum SLR levels in SLE-patients and their autoantibody titers, socio-demographic, psychological, environmental, dietary, laboratory, smoking and lifestyle parameters all of which were intelligently defined as quantitative values.

After putting together all the correlations, it could be expected that depression at any level can be harmful for SLE-patients, so that increasing the BDI score as a risk factor may be associated with the occurrence of lupus disease. Moreover, decreased serum concentration of SLR in these patients may be implicated in the pathogenesis of lupus.
